# Multi-type *RFC1* repeat expansions as the most common cause of hereditary sensory and autonomic neuropathy

**DOI:** 10.3389/fneur.2022.986504

**Published:** 2022-08-17

**Authors:** Jun-Hui Yuan, Yujiro Higuchi, Masahiro Ando, Eiji Matsuura, Akihiro Hashiguchi, Akiko Yoshimura, Tomonori Nakamura, Yusuke Sakiyama, Jun Mitsui, Hiroyuki Ishiura, Shoji Tsuji, Hiroshi Takashima

**Affiliations:** ^1^Department of Neurology and Geriatrics, Kagoshima University Graduate School of Medical and Dental Sciences, Kagoshima, Japan; ^2^Department of Molecular Neurology, Graduate School of Medicine, The University of Tokyo, Tokyo, Japan; ^3^Department of Neurology, Faculty of Medicine, The University of Tokyo, Tokyo, Japan; ^4^Institute of Medical Genomics, International University of Health and Welfare, Chiba, Japan

**Keywords:** *RFC1*, sensory neuropathy, non-coding repeat expansion, gene panel sequencing, haplotype

## Abstract

Non-coding repeat expansions within *RFC1* and *NOTCH2NLC* genes have lately been linked to multisystem neurodegenerative diseases, which also shed light on yet undiagnosed patients with inherited peripheral neuropathies. The aim of this study was to identify the genetic basis of patients with hereditary sensory and autonomic neuropathy (HSAN). We collected 79 unrelated DNA samples clinically suspected with HSAN from multiple regions of Japan. Mutation screening was first performed using gene panel sequencing and whole-exome sequencing. Pathogenic/likely pathogenic variants were identified from genes of *WNK1/HSN2* (6 cases), *SCN9A* (3 cases), *NTRK1* (3 cases), and *DNMT1* (2 cases). Subsequently, long-range flanking PCR and repeat-primed PCR were applied to analyze repeat expansions in *RFC1* and *NOTCH2NLC*. Bi-allelic *RFC1* repeat expansions were detected from 20 adult-onset HSAN patients, consisting of [(AAGGG)exp/(AAGGG)exp] (8 cases), [(ACAGG)exp/(ACAGG)exp] (8 cases), and [(AAGGG)exp/(ACAGG)exp] (4 cases). GGC repeat expansion in *NOTCH2NLC* was found in 1 case. Single-nucleotide variant-based haplotype analysis of patients harboring disease-associated repeat expansions in *RFC1* revealed distinguishable haplotypes among subgroups with different repeat genotypes. These findings substantially redefine the genetic spectrum of HSAN, where multi-type *RFC1* repeat expansions account for 25.3% of all patients, highlighting the necessity of genetic screening, particularly for adult-onset patients.

## Introduction

Hereditary sensory and autonomic neuropathy (HSAN) or hereditary sensory neuropathy (HSN) is a group of rare inherited disorders, characterized by severe loss of pain and other sensations, with/without autonomic nervous dysfunctions. To date, approximately 20 genes have been linked to the HSAN phenotype (https://neuromuscular.wustl.edu/). Nevertheless, a comprehensive genetic spectrum study of HSAN is rare, and genetic screening could only yield a diagnostic rate of 14.3–35% (1–3).

In 2019, a bi-allelic expansion of intronic pentanucleotide repeat AAGGG was identified in the *RFC1* gene, from patients with late-onset ataxia, accompanied by proprioceptive and vestibular impairment, thus also known as cerebellar ataxia, neuropathy, vestibular areflexia syndrome (CANVAS) (4, 5). Thereafter, another pathogenic pentanucleotide repeat, ACAGG, was uncovered from multiple Asia-Pacific regions (6, 7). *RFC1*-related clinical spectrum has been expanded, including, but not limited to, multiple system atrophy, sensory-pure neuropathy, and sensory-predominant neuropathy (8–12). Also in 2019, we demonstrated the association between a GGC repeat expansion of 5'-untranslated region (UTR) in the *NOTCH2NLC* gene and neuronal intranuclear inclusion disease (NIID), characterized by eosinophilic intranuclear inclusions in the nervous system, skin, and visceral organs (13–15). Clinically, these patients manifest a broad spectrum of nervous system phenotypes, including dementia, Parkinsonism, tremor, cerebellar ataxia, seizure, peripheral neuropathy, and autonomic symptoms (14–16).

Given that sensory neuropathy may present as the original or predominant phenotype of *RFC1/NOTCH2NLC*-related disorders, we screened these non-coding repeat expansions in a Japanese case series with HSAN. Together with our preceding HSAN-related gene panel sequencing and whole exome sequencing (WES), these findings enable us to update the genetic spectrum of HSAN.

## Methods

### Enrollment criteria

From 2004 to 2021, we consecutively collected DNA samples of 79 unrelated patients from extensive regions of Japan, who were clinically suspected with HSAN/HSN. Clinical enrollment criteria were defined as (i) pure/predominant sensory symptoms (loss of pain/temperature sensation; paraesthesia) and/or apparent dysautonomia; and (ii) pure sensory or sensory-dominant polyneuropathy, revealed by nerve conduction study (NCS).

This study was approved by the institutional review board of Kagoshima University (Application ID: 490). All patients/parents and their available family members provided informed consent for participation in this study.

### Gene panel sequencing and WES

Mutation screening was first performed by next-generation sequencing (NGS)-based gene panel analysis, targeting 18 HSAN-related genes (Supplementary Table 1). Library preparation and sequencing using the Illumina MiSeq platform (Illumina Inc., San Diego, California) were conducted according to a previously described workflow (17). Data alignment and variant processing were performed *via* CLC Genomics Workbench (QIAGEN, Hilden, Germany).

Subsequently, WES was carried out on 25 among 65 undiagnosed cases. Exome sequence was enriched using SureSelect v4+UTRs or v5+UTRs Kit (Agilent Technologies, CA, USA) and sequenced on Hiseq2000/Hiseq2500 platform (Illumina Inc., San Diego, CA, USA) at Tokyo University. Sequencing data were aligned to human genome data (GRCh37/hg19), and variant calling was performed using Burrows-Wheeler Aligner and SAM tools (18). Variants of VCF files were analyzed using Ensembl-VEP and our in-house R script pipeline. (The in-house R scripts are available online at https://github.com/jhyuans/hsan.)

All genomic variants were checked against the public population databases, including Genome Aggregation Database (gnomAD v2.1.1; https://gnomad.broadinstitute.org) and Japanese Multi Omics Reference Panel (jMorp; https://jmorp.megabank.tohoku.ac.jp/202102/) (19), an in-house WES database, and Human Gene Mutation Database (HGMD 2021.3, QIAGEN). Multiple *in silico* prediction scores were enrolled, comprising SIFT, PolyPhen2, PROVEAN, FATHMM, and Condel (20). All candidate variants were validated using Sanger sequencing and classified following a modified guideline of the American College of Medical Genetics and Genomics/Association for Molecular Pathology (ACMG/AMP) and ClinGen Expert Panel consensus methods (21, 22) (Supplementary Table 2).

### Long-range flanking PCR and repeat-primed PCR (RP-PCR) of *RFC1* repeat expansions

Long-range flanking PCR was applied for the preliminary screening of all 79 samples, using primers flanking the *RFC1* intronic repeat expansion locus and a modified protocol referring to a previous report (7). The samples that failed to yield a clear PCR product, suggesting large homozygous expansion, were processed through RP-PCR. Four types of FAM-labeled repeated primer sets were applied, respectively, targeting AAAAG (reference allele), AAAGG (benign variant), AAGGG (pathogenic), and ACAGG (pathogenic) expansions (Supplementary Table 3).

### RP-PCR and amplicon length analysis of *NOTCH2NLC*

All undiagnosed HSAN cases through NGS were processed through (GGC)exp analysis in the *NOTCH2NLC* gene. Primer design, RP-PCR, and fluorescence amplicon length analysis were carried out according to our previous study (14).

All PCR products of *RFC1* and *NOTCH2NLC* were subjected to capillary electrophoresis using the ABI PRISM 3130xL Genetic Analyzer (Applied Biosystems, Foster City, CA, USA), and results were visualized using the Peakscanner software (Applied Biosystems).

### Single-nucleotide variant (SNV)-based haplotype analysis around *RFC1*

In total, 18 SNVs spanning the repeat expansion locus in *RFC1*, applied by previous reports or detected by our WES, were enrolled to construct the homologous haplotype. This haplotype was then compared with the core disease-related haplotype identified in Caucasian patients with CANVAS (5).

### Pathological study of peripheral nerve

To analyze the pathological and morphometric changes of the sensory nerve, sural nerve biopsies were performed. Semithin sections from Epon embedded tissues were prepared and stained with toluidine blue. Images were acquired using a light microscope (OLYMPUS DP27, Olympus Corporation, Japan). Myelinated nerve fiber density was calculated with the Luzex AP image analysis software (Nireco Corp., Tokyo, Japan).

### Statistical analysis

Statistical differences were calculated using the Student's *t*-test or one-way ANOVA test for quantitative data (onset age) among subgroups of patients. Fisher's exact test was applied for categorical data whenever available. All analyses and plotting were performed using R studio (Version 1.4), and a *p*-value of less than 0.05 was considered significant.

## Results

### Genetic findings

**Figure 1:**
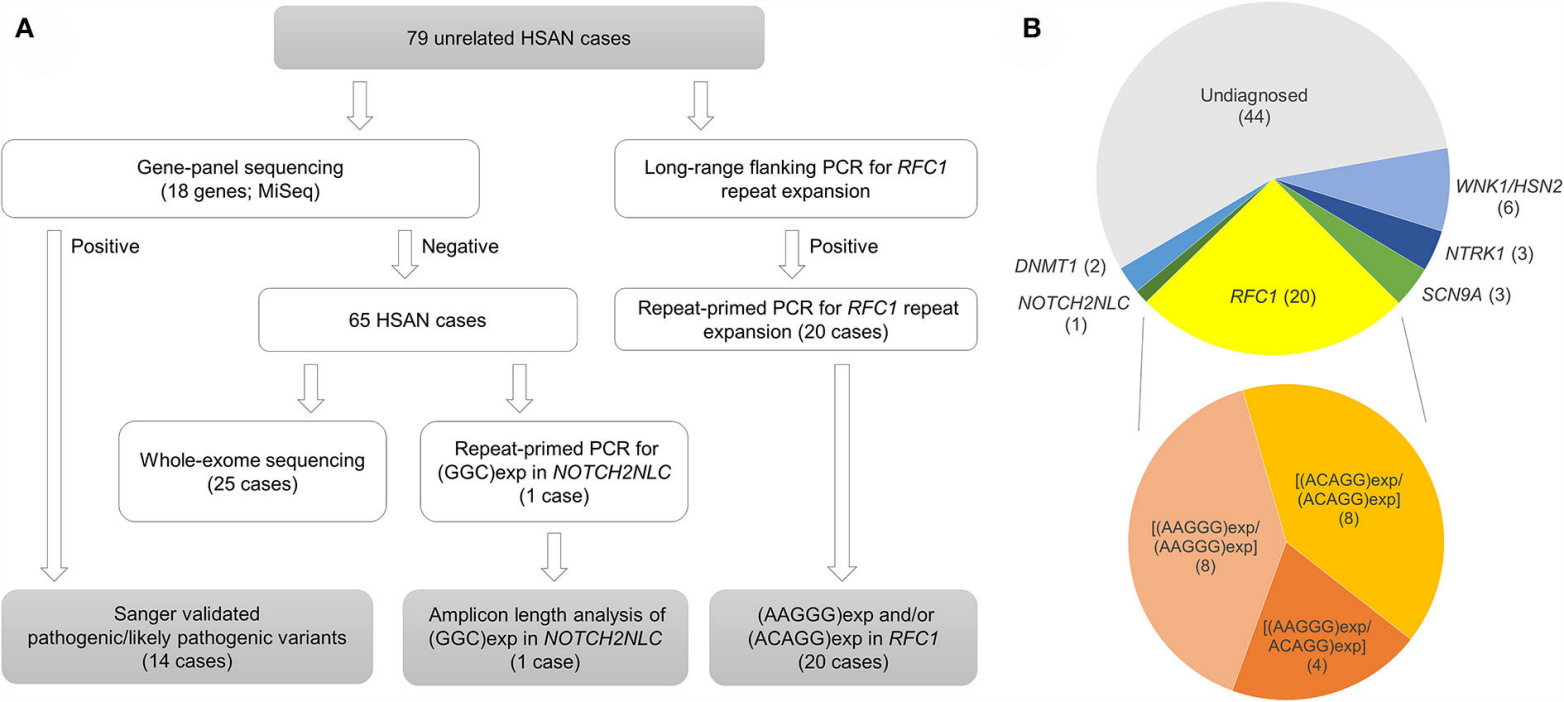
Genetic analysis workflow **(A)** and proportional piechart of genes with pathogenic/likely pathogenic variants from diagnosed HSAN patients **(B)**.

The genetic analysis workflow is summarized in Figure 1A. Among 79 patients with HSAN in our case series, gene panel sequencing revealed pathogenic/likely pathogenic variants from 14 cases in four genes, namely, *WNK1/HSN2* (6 cases), *NTRK1* (3 cases), *SCN9A* (3 cases), and *DNMT1* (2 cases) (Figure 1B; Table 1). Part of these patients and their variants in *WNK1/HSN2* (5 cases), *SCN9A* (2 cases), and *DNMT1* (1 case) had been reported by our group previously (3, 17, 23). Detailed application of ACMG/AMP guidelines for these variants is listed in Supplementary Table 4.

**Table 1:** All pathogenic/likely pathogenic variants detected by genetic studies and clinical features of our patients.

**Gene**	**Nucleotide**	**Protein**	**Genotype**	**Cases**	**ACMG**	**Onset age**	**Clinical features**
*WNK1/HSN2*	c.3237dup	p.Asp1080*	HO	4	P	Congenital~ adolescent	Early-onset severe distal loss of pain and temperature sensation; recurrent ulcero-mutilation in hands and feet; dyshidrosis
	c.3237dup+c.2615C>G	p.Asp1080*+p.Ser872*	CH	1	P/P		
	c.3237dup+c.2971C>T	p.Asp1080*+p.Arg991*	CH	1	P/P		
*NTRK1*	c.1642del+c.2002G>T	p.Arg548Glyfs*104+p.Asp668Tyr	CH	1	P/P	Congenital	Loss of pain and temperature sensation; recurrent ulcer; anhidrosis; mental retardation; thermal dysregulation; joint deformities
	c.1642del+c.1786C>T	p.Arg548Glyfs*104+p.Arg596*	CH	1	P/P		
	c.1642del+c.2285C>T	p.Arg548Glyfs*104+p.Pro762Leu	CH	1	P/LP		
*SCN9A*	c.3993delinsTT	p.Leu1331Phefs*8	HO	2	P	Congenital~ adolescent	Loss of pain and temperature sensation; hypohidrosis; hyposmia; hearing loss; bone dysplasia
	c.4895C>A	p.Ala1632Glu	HE	1	P	Congenital	Paroxysmal apnea, hypoxemia, and bradycardia with facial erythema; gastroesophageal reflux; delayed motor milestone
*DNMT1*	c.1706A>G	p.His569Arg	HE	1	LP	Adolescent~adult	Severe loss of pain and temperature sensation; recurrent ulcero-mutilation; sensorineural hearing loss; early-onset dementia; cerebellar ataxia and atrophy
	c.1619A>G	p.Tyr540Cys	HE	1	P		
*NOTCH2NLC*	(GGC)exp		HE	1	P	Adolescent	Tremor; recurrent vomiting; muscle weakness; dementia; paresthesia; dysphagia; dysarthria; orthostatic hypotension; hyperhidrosis
*RFC1*	[(AAGGG)exp/(AAGGG)exp]		HO	8	P	Middle-aged adult~	Paresthesia/numbness; ataxia; dysautonomia; dysarthria/dysphagia; eye movement disorders; chronic cough
	[(ACAGG)exp/(ACAGG)exp]		HO	8	LP		
	[(AAGGG)exp/(ACAGG)exp]		CH	4	P/LP		

HO, homozygous; CH, compound heterozygous; HE, heterozygous; P, pathogenic; LP, likely pathogenic.

**Figure 2:**
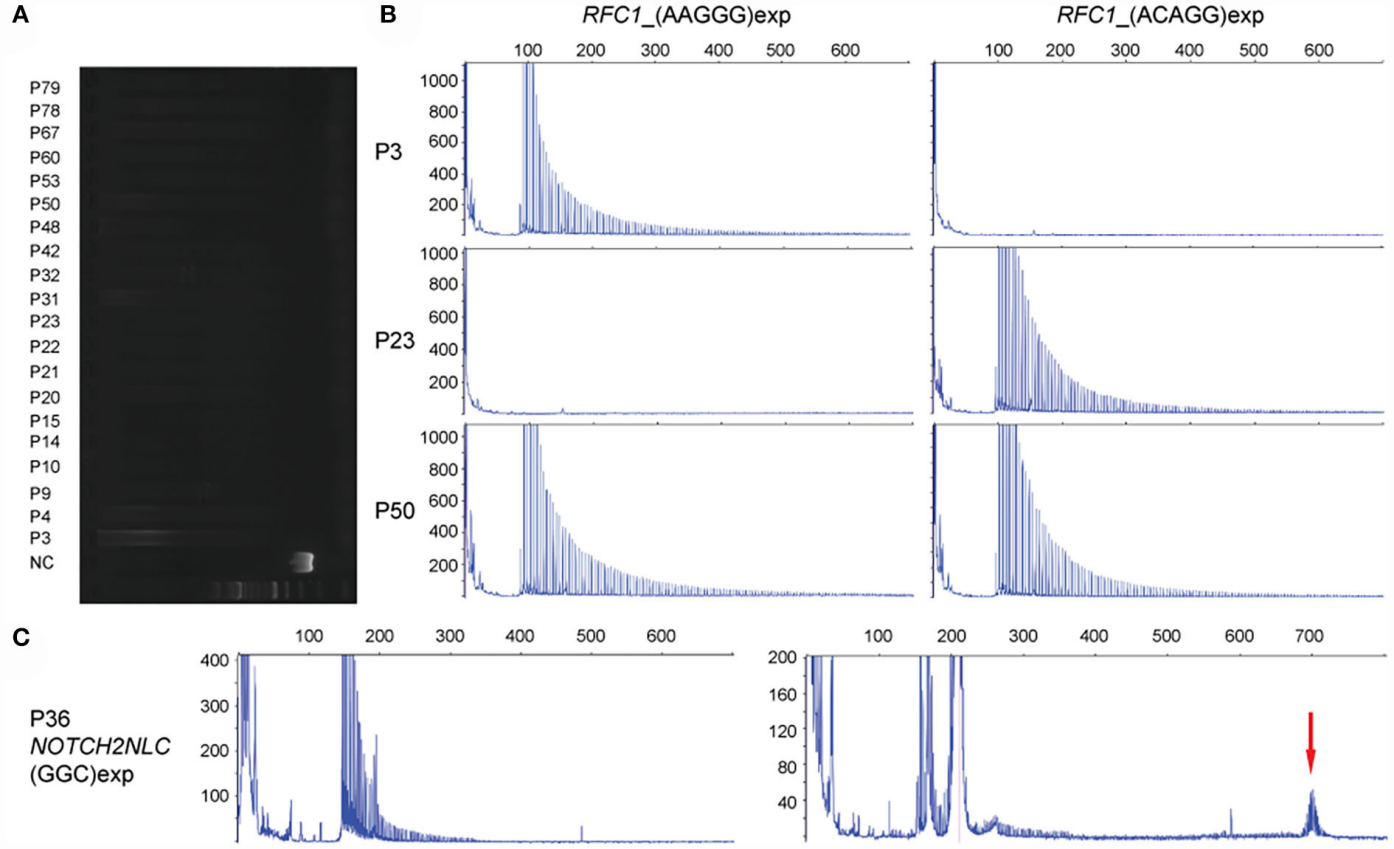
Detection of disease-associated repeat expansions in *RFC1* and *NOTCH2NLC* genes. **(A)** Long-range PCR shows the absence of clear amplifiable PCR product in *RFC1* from 20 cases with HSAN. NC: negative control. **(B)** Examples of [(AAGGG)exp/(AAGGG)exp] (P3), [(ACAGG)exp/(ACAGG)exp] (P23), and [(AAGGG)exp/(ACAGG)exp] (P50) of *RFC1*, in the decremental saw-tooth pattern. **(C)** Repeat-primed PCR reveals (GGC)exp in *NOTCH2NLC* (P36), and amplicon length is determined by fluorescence amplicon length analysis (red arrow).

Disease-associated repeat expansion in *RFC1* was identified from 20 cases (13 males and 7 females), referring to the following criteria: (1) absence of clear PCR-amplifiable product on flanking PCR; (2) absence of smear PCR product after RP-PCR showing for (AAAAG)exp (reference allele) or (AAAGG)exp (benign allele); (3) presence of a decremental saw-tooth pattern on Peakscanner of RP-PCR product for (AAGGG)exp or/and (ACAGG)exp. Homozygous [(AAGGG)exp/(AAGGG)exp] and [(ACAGG)exp/(ACAGG)exp] were detected from 8 cases each, respectively. Additionally, from the other 4 cases, both (AAGGG)exp and (ACAGG)exp alleles were identified (Figures 2A,B). We did not find any other suspected bi-allelic variants in these 20 cases, regardless of using gene panel sequencing or WES. Otherwise, GGC repeat expansion in *NOTCH2NLC* was detected from one patient (P36) and the repeat number was 113 (Figure 2C).

### Clinical summary of patients with disease-associated *RFC1* repeat expansions

The mean age of 20 patients was 68.2 ± 8.4 years, and their mean age of onset was 57.8 ± 12.0 years; 11 cases were sporadic (9/18), and the other seven cases were suspected with autosomal recessive inheritance, on account of their consanguineous parents or affected siblings. Most common original symptom was paresthesia/numbness (*n* = 10), followed by walking/standing unsteadiness (*n* = 7), dysarthria (*n* = 1), dysuria (*n* = 1), and muscle cramp (*n* = 1). Sensory dysfunctions were verified from all 20 cases and, more specifically, from pain sensation (*n* = 17), vibration sensation (*n* = 17), or position sensation (*n* = 7). Various autonomic dysfunctions were recorded from 10 cases, mainly comprising urination disorders (*n* = 7), constipation (*n* = 6), and orthostatic hypotension (*n* = 4). Muscle weakness/atrophy was noted from 7 cases, involving interosseus muscle (*n* = 4), left hamstrings muscle (*n* = 1), gastrocnemius muscle (*n* = 1), or flexion/extension of shoulder and hip joint (*n* = 1). There were 11/20 cases showing signs of ataxia, and Romberg's sign was found positive in 14/17 cases. Hearing loss was identified in 4/15 cases, and the vestibular function test was carried out only on P53, suggesting vestibular hypofunction. Additionally, 3/15 cases had a record of chronic cough history, which could precede the onset of neurological symptoms. All clinical features are shown in Table 2 and Supplementary Table 5.

**Table 2:** Clinical features of 20 cases with multi-type disease-associated *RFC1* repeat expansions.

	**All cases** **(*n* = 20)**	**[(ACAGG)exp/ (ACAGG)exp] (*n* = 8)**	**[(AAGGG)exp/ (ACAGG)exp] (*n* = 4)**	**[(AAGGG)exp/ (AAGGG)exp] (*n* = 8)**	**[(AAGGG)exp/ (AAGGG)exp] (Currò R, et al. *n* = 43)**	***p* value**
Gender (male/total)	13/20	6/8	1/4	6/8	25/43	/
Mean age at onset (years)	57.8 ± 12.0	55.4 ± 13.2	58.2 ± 10.7	59.9 ± 12.3	56 (30~75)	/
Reduced pain sensation	17/20	7/8	4/4	6/8	28/43	>0.05
Reduced vibration sensation	17/20	7/8	3/4	7/8	35/43	>0.05
Reduced position sensation	7/20	3/8	2/4	2/8	10/43	>0.05
Dysautonomia	10/17	4/7	2/4	4/6	9/43	**0.036**
Muscle weakness/atrophy	7/19	3/7	2/4	2/8	0/43	**0.022**
Reduced tendon reflex	11/18	4/7	3/4	4/7	27/43	>0.05
Eye movement disorder	7/18	4/7	0/3	3/8	20/40	>0.05
Dysarthria/dysphagia	7/18	2/7	0/3	5/8	10/43	**0.039**
Ataxia	11/20	5/8	2/4	4/8	32/43	>0.05
Positive Romberg sign	14/17	6/6	2/4	6/7	22/43	>0.05
Chronic cough	3/15	1/6	0/3	2/6	26/43	>0.05
Cerebellar atrophy (MRI)	8/14	5/7	0/1	3/6	7/27	>0.05

/: not available. All p-values < 0.05 are indicated in bold.

**Figure 3:**
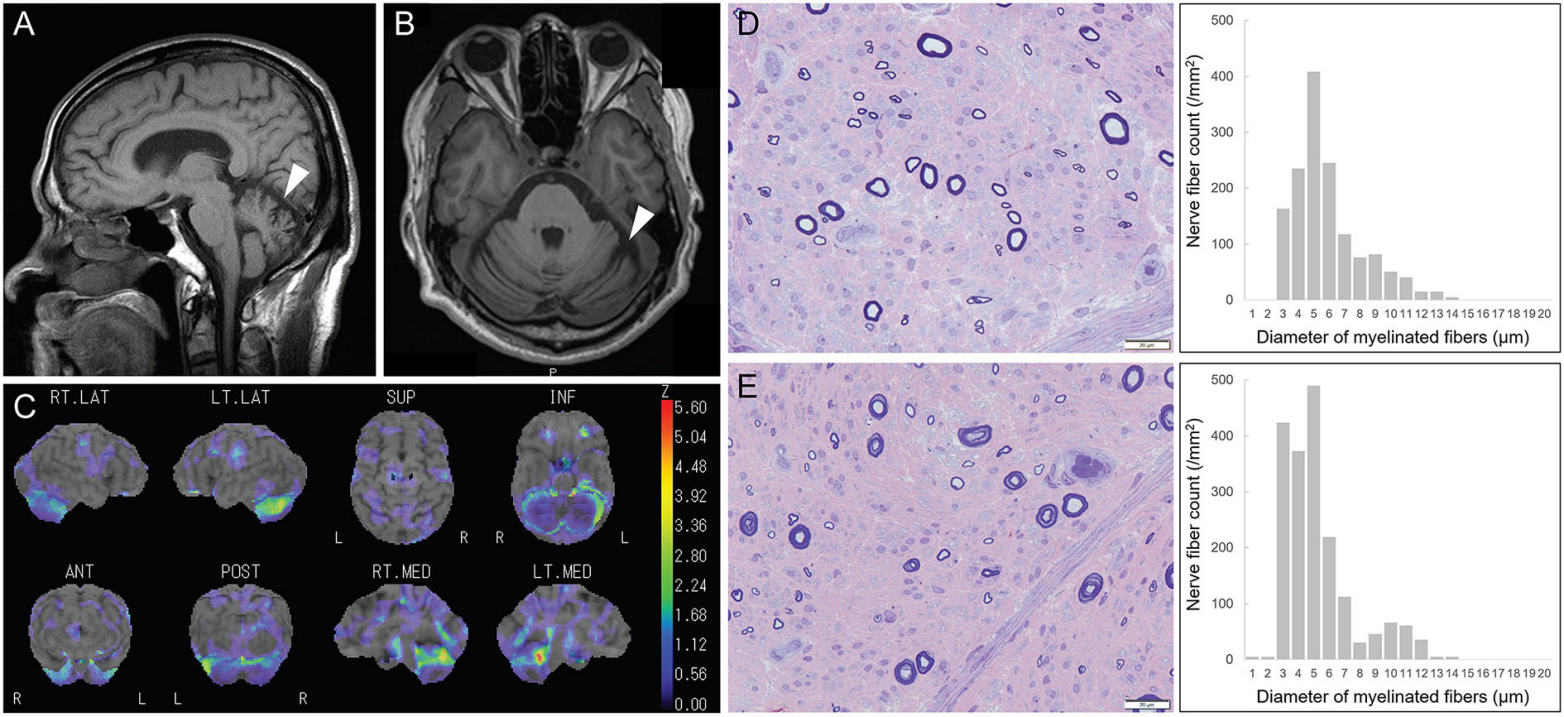
Radiological and pathological features of patients with disease-associated *RFC1* repeat expansions. **(A,B)** Brain MRI shows cerebellar atrophy (white arrow; P10). **(C)** Brain single photon emission computed tomography (SPECT) reveals decreased blood flow in the cerebellar region (P21). **(D,E)** Marked loss of large and small myelinated fibers (large > small). Myelinated fiber densities are 1,456/mm^2^ and 1,880/mm^2^, respectively, from P31 with [(AAGGG)exp/(AAGGG)exp] and P10 with [(ACAGG)exp/(ACAGG)exp].

MRI data were available from 14 cases, which revealed atrophy of the cerebellum (*n* = 8), spinal cord (*n* = 2), and frontal lobe (*n* = 1) (Figure 3A; P10). Using brain single photon emission computed tomography (SPECT), decreased blood flow was recognized in 4/5 of cases, mainly in the cerebellar regions (*n* = 3) (Figure 3B; P21).

Using NCS, sensory nerve action potentials of 14 patients, regardless of upper or lower limbs, were found not evoked or reduced remarkably. On the contrary, in the other five cases, sensory nerve impairment was found more severe in their lower limbs than that in their upper limbs. Motor nerve conduction studies were normal in all but P60, showing mildly decreased compound muscle action potentials and motor conduction velocities of the right tibial nerve. All patients were electrophysiologically comparable with predominant sensory axonal neuropathy. Sensory-evoked potential study revealed prolonged latency (*n* = 4) or not evoked (*n* = 1) among six available cases (Supplementary Table 5).

### SNV-based homologous haplotype around *RFC1*

Haplotype was originally inferred using genomic variants obtained from 11/20 patients with available WES data, including 6 cases with (AAGGG)exp, 4 cases with (ACAGG)exp, and 1 case harboring both. We reconstructed the “core haplotype” region on the basis of Caucasian CANVAS patients, spanning from chr4:38995374 (rs10212770) to chr4:39448586 (rs35372803) (5). In total, we enrolled 14 previously described SNVs, and four new SNV markers, namely, rs2066789 (39308187G>A), rs947301858 (39324897C>T), rs3736168 (39368083C>T), and rs900563 (39409416C>A).

**Figure 4:**
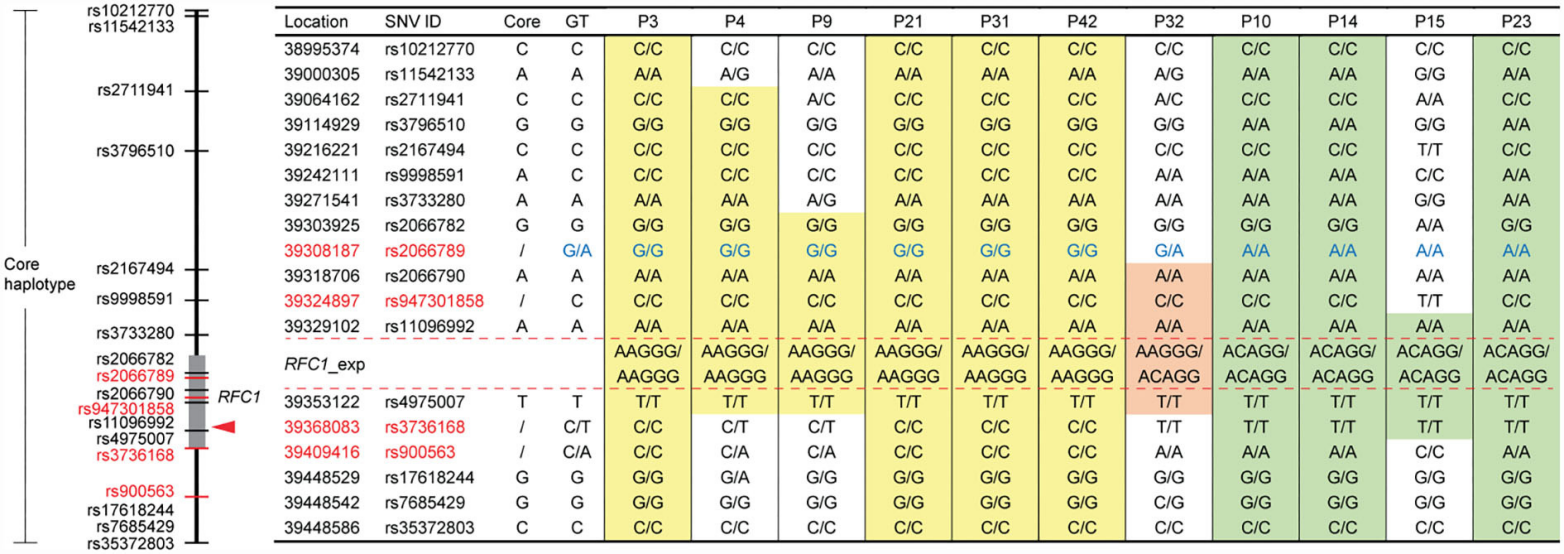
Single-nucleotide variant (SNV)-based haplotype analysis of 11 cases with different disease-associated *RFC1* repeat expansions. The “core haplotype” region, spanning from chr4:38995374 (rs10212770) to chr4:39448586 (rs35372803), is covered by 14 previously applied and 4 new SNV markers (red color). GT: genotype. Respectively, 49.2 kb (chr4: 39303925~39353122) and 453.2 kb (chr4: 38995374~39448586) highly conserved homologous haplotype blocks are identified from all six cases carrying [(AAGGG)exp/(AAGGG)exp] (yellow background) and 3/4 of cases with [(ACAGG)exp/(ACAGG)exp] (green background). P32 carrying [(AAGGG)exp/(ACAGG)exp] shares an identical haplotype block (chr4: 39318706~39353122; light red background) with other repeat expansion subtypes. Genotype of rs2066789 (G/A) is haplotype specific (blue color), and the haplotype of P15 is unique.

All 6 cases, harboring [(AAGGG)exp/(AAGGG)exp], were found to share a shorter “core haplotype,” ranging from rs2066782 to rs4975007 (49.2 kb), whereas the CC at rs9998591 was different from previous reports in Caucasian patients (5). Among 4 cases with [(ACAGG)exp/(ACAGG)exp], except for P15, the other 3 cases share an identical haplotype in this whole region (453.2 kb). A haplotype-specific variant for [(ACAGG)exp/(ACAGG)exp], rs2066789-AA, was identified, which was then validated in the other cases without WES data using Sanger sequencing (Data not shown). Intriguingly, we also found a unique haplotype in P15, which was distinguishable from any of the other patients, including a rare variant, rs947301858-TT, between rs2066790 and rs11096992 (Figure 4).

### Sural nerve pathology

Sural nerve biopsy was performed in 11 cases with disease-associated *RFC1* repeat expansions. All sural nerve pathology showed marked chronic loss of both large and small myelinated fibers, without active axonal degeneration, demyelination, or regeneration. The pathological findings of cases with [(AAGGG)exp/(AAGGG)exp] and [(ACAGG)exp/(ACAGG)exp] were comparable (Figures 3D,E).

### Statistical analysis for patients with disease-associated *RFC1* repeat expansions

No significant difference was identified in age at onset between male (61.2 ± 11.6 years) and female (51.4 ± 10.6 years) patients (Student's *t*-test, *p* = 0.08). Among three subgroups harboring distinct genotypes, there was no significant difference in terms of either their age at onset or any of the clinical features (Table 2). In comparison with a recent study of European patients with idiopathic sensory neuropathy and [(AAGGG)exp/(AAGGG)exp] in *RFC1* (8), our patients carrying [(AAGGG)exp/(AAGGG)exp] showed significantly higher frequency (Fisher's exact test) of motor weakness/atrophy (*p* = 0.022), dysautonomia (*p* = 0.036), and dysarthria/dysphagia (*p* = 0.039) (Table 2; gray background).

## Discussion

Identification of non-coding repeat expansions in *RFC1* and *NOTCH2NLC* genes enables us to update the genetic spectrum of HSAN and remarkably increases our diagnostic rate. Multi-type bi-allelic pentanucleotide repeat expansion in *RFC1* is the most common causative reason in our HSAN case series (25.3%), far ahead of the following genes, including *WNK1/HSN2* (7.6%), *NTRK1* (3.8%), *SCN9A* (3.8%), *DNMT1* (2.5%), and *NOTCH2NLC* (1.3%). Patients harboring pathogenic *RFC1* repeat expansions share middle-aged adult-onset (age: 35–78 years) sensory predominant neuropathy, frequently accompanied by various autonomic and cerebellar dysfunctions. They are generally differentiable in the clinic against patients carrying pathogenic/likely pathogenic variants within other HSAN disease-causing genes.

Most recently, two European groups demonstrated that bi-allelic [(AAGGG)exp/(AAGGG)exp] of *RFC1* account for 30–34% of their patients with pure sensory ataxic neuropathy or idiopathic axonal polyneuropathy (8, 24). Our findings further supported their observation, albeit not as high as their prevalence, and validated its commonness not only in European studies but also in Asian studies. The importance of this similarity ought to be highlighted considering the well-recognized prevalence difference of repeat expansion diseases, such as Friedreich's ataxia and Dentatorubral-pallidoluysian atrophy, between Asian and European studies (25, 26).

Clinically, predominant sensory dysfunctions, particularly in pain and vibration sensations, together with severe sensory nerve impairment revealed by NCS, suggest the diagnosis of HSAN, although cerebellar ataxia and atrophy were noted in 11/20 cases as well; 25% of our patients with [(AAGGG)exp/(AAGGG)exp] developed mild muscle weakness, more frequently in distal muscles, which is significantly different from the European study (*p* < 0.05) (8), but echo to another study in Italy, where (AAGGG)exp was also detected in patients with sensorimotor neuropathy (10), as well as our in-house observation (paper in press). Taken together, *RFC1* repeat expansion screening should also be recommended for the most common inherited peripheral neuropathy, hereditary motor and sensory neuropathy, or Charcot-Marie-Tooth disease.

Using long-range franking PCR and RP-PCR, we identified bi-allelic [(AAGGG)exp/(AAGGG)exp] and [(ACAGG)exp/(ACAGG)exp] in *RFC1* from eight patients each, respectively. They enable us to investigate the correlation between two genotypes and their clinical phenotypes from a group of patients with identical genetic background. Despite the onset age of patients harboring [(ACAGG)exp/(ACAGG)exp] seems younger than that of patients with [(AAGGG)exp/(AAGGG)exp] (55.4 ± 13.2 years vs. 59.9 ± 12.3 years), no significant difference was found in terms of their onset age nor from other clinical features. Additionally, another disease-associated genotype in *RFC1*, compound heterozygous [(AAGGG)exp/(ACAGG)exp], was demonstrated in four HSAN patients. Clinical features of these patients were found comparable to other patients with homozygous expansions in *RFC1*.

Founder mutations, like p.Asp1080^*^ in *WNK1/HSN2* (6 cases) and p.Arg548Glyfs^*^1 in *NTRK1* (3 cases), have been verified previously by us and another team, respectively (3, 27), suggesting that it is a relatively common phenomenon in Japan. With respect to *RFC1*, using WES and SNV-based haplotype analysis, we identified a shorter version of the “core haplotype” (49.2 kb; chr4: 39303925~39353122), shared by all 6 cases with homozygous [(AAGGG)exp/(AAGGG)exp]. Meanwhile, a much longer homologous haplotype (453.2 kb; chr4: 38995374~39448586) was found completely conserved among 3/4 of cases harboring homozygous [(ACAGG)exp/(ACAGG)exp], suggesting a strong founder effect. Notably, this haplotype is distinguishable from the (AAGGG)exp-containing haplotype, and the (ACAGG)exp-specific variant, rs2066789-AA, might become a useful haplotype indicator. An entirely different origin is speculated due to the unique haplotype of P15 with [(ACAGG)exp/(ACAGG)exp]. These findings sustain the importance of refined haplotype analysis for further understanding the ancestral origin of disease-associated repeat expansions. A recent study of healthy Japanese individuals revealed that 22/281 (7.8%) and 0/276 (0%) participants had AAGGG and ACAGG expansions, respectively. Our haplotype analysis further supported their speculation that the (AAGGG)exp haplotype arose earlier and the (ACAGG)exp haplotype diverged from it (28).

Unlike (AAGGG)exp of *RFC1*, (GGC)exp in *NOTCH2NLC* has been found rare in European patients with movement disorders, essential tremor, or leukoencephalopathy (29–31). (GGC)exp in *NOTCH2NLC* could lead to gain-of-function effects, inducing cytotoxicity and impairing nucleocytoplasmic transport, a cellular process associated with aging and neurodegeneration (32). *RFC1* encodes the large subunit of replication factor C (RFC) and plays a key role in DNA damage recognition and recruitment of DNA-repair enzymes (4). Despite its homozygous inheritance pattern suggesting a loss-of-function mechanism, further analyses of (AAGGG)exp in *RFC1* did not reveal any notable difference in expression level or aberrant splicing events, nor evident different responses to physical/chemical factors for DNA impairment (4, 5). Intron 2 retention in *RFC1* pre-mRNA across various tissues has been identified in patients, which is known as a common event associated with other disease-causing guanine-cytosine-rich intronic expansions, like myotonic dystrophy type 2 and C9orf72-amyotrophic lateral sclerosis (4).

In summary, the identification of bi-allelic disease-associated repeat expansions in *RFC1* fundamentally changes the genetic spectrum of HSAN and raises the overall diagnostic rate up to 44.3%. Although no significant clinical difference was observed among our cases with diverse repeat genotypes, a larger study with more samples would be helpful to further understand their genotype–phenotype association. The [(AAGGG)exp/(ACAGG)exp] genotype, the unique haplotype of P15, and distinguishable haplotypes for both (AAGGG)exp and (ACAGG)exp underline the racial diversities and enrich the ancestral complexity of disease-associated *RFC1* repeat expansions. Our findings would be beneficial for the neurologist to better understand the genetic basis of HSAN and for establishing a more efficient genetic screening strategy in the clinic.

## Data availability statement

The datasets presented in this article are not readily available because of ethical and privacy restrictions. Requests to access the datasets should be directed to the corresponding author/s.

## Ethics statement

The studies involving human participants were reviewed and approved by the Institutional Review Board of Kagoshima University (Application ID: 490). Written informed consent to participate in this study was provided by the participants' legal guardian/next of kin.

## Author contributions

HT conceived the project and designed the study. J-HY and AY conducted the genetic experiments and analyzed the data. J-HY, YH, MA, AH, and YS participated in clinical data acquisition and analysis. EM and TN contributed to pathological and electrophysiological analyses, respectively. JM, HI, and ST contributed to whole-exome sequencing and data processing. J-HY drafted the original manuscript. All authors approved the final version.

## Funding

This study was supported by Grants-in-Aid from the Research Committee of Ataxia, Health Labor Sciences Research Grant, and the Ministry of Health, Labor and Welfare, Japan (20317603 and 201610002B). This research was also supported by the Research Program for Conquering Intractable Disease from Japan Agency for Medical Research and Development (AMED) (201442014A and 201442071A) and JSPS KAKENHI Grants (JP18H02742, JP20K16604, JP21K15702, JP21H02842, JP22K15713, and JP22K07495).

## Conflict of interest

The authors declare that the research was conducted in the absence of any commercial or financial relationships that could be construed as a potential conflict of interest.

## Publisher's note

All claims expressed in this article are solely those of the authors and do not necessarily represent those of their affiliated organizations, or those of the publisher, the editors and the reviewers. Any product that may be evaluated in this article, or claim that may be made by its manufacturer, is not guaranteed or endorsed by the publisher.
