# Supplementary material for: Multi-type RFC1 repeat expansions as the most common cause of hereditary sensory and autonomic neuropathy
Source: Front Neurol. 2022 Aug 17;13:986504. doi: 10.3389/fneur.2022.986504 (PMC9428154; doi:10.3389/fneur.2022.986504)
Supplement: Supplementary file 1 [file Data_Sheet_1.PDF]

Supplementary Table 1 18 genes involved in our HSAN-related gene panel sequencing

Gene symbol	Transcript
ATL1	ENST00000441560
ATL3	ENST00000398868
CCT5	ENST00000280326
DNMT1	ENST00000359526
DST	ENST00000244364
FAM134B	ENST00000306320
FLVCR1	ENST00000366971
IKBKAP	ENST00000374647
KIF1A	ENST00000498729
NGF	ENST00000369512
NTRK1	ENST00000368196
PRNP	ENST00000379440
RNF170	ENST00000527424
SCN11A	ENST00000302328
SCN9A	ENST00000303354
SPTLC1	ENST00000262554
SPTLC2	ENST00000216484
WNK1/HSN2	ENST00000537687

Supplementary table 2 Modified ACMG/AMP classification

Evidence class	ACMG criteria	Comment	Classification
PVS1	Null variant (nonsense, frameshift, canonical +/− 1 or 2 splice sites, initiation codon, single or multi-exon deletion) in a gene where loss of function (LOF) is a known mechanism of disease	Follows primary definition in the following genes: *WNK1/HSN2, SCN9A, NTRK1*	PVS
PS1	Same amino acid change as a previously established pathogenic variant regardless of nucleotide change.	Follows primary definition but none of the variants meet this criterion	Not applicable
PS2/PM6	PS2: De novo (both maternity and paternity confirmed) in a patient with the disease and no family history<br>PM6: Assumed de novo, but without confirmation of paternity and maternity	Follows primary definition	PM6
PS3	Well-established in vitro or in vivo functional studies strongly supportive of a damaging effect on gene or gene product	Follows primary definition	PS3
PS4	The prevalence of the variant in affected individuals is significantly increased compared to the prevalence in controls	Refers to "ClinGen's RASopathy Expert Panel consensus methods for variant interpretations"	PS4: ⩾5 independent occurrences<br>PS4(M): 3~4 independent occurrences<br>PS4(P): 1~2 independent occurrences
PM1	Located in a mutational hot spot and/or critical and well-established functional domain (e.g. active site of an enzyme) without benign variation	Follows primary definition but none of the variants met this criterion.	Not applicable
PM2	Absent from controls (or at extremely low frequency if recessive) in Exome Sequencing Project, 1000 Genomes or ExAC	Completely absent in both East Asian population of gnomAD and jMorp databases	PM2
PM3	For recessive disorders, the variant is detected in trans with a pathogenic variant.	Follows primary definition	PM3
PM4	Protein length changes as a result of in-frame deletions/insertions in a nonrepeat region or stop-loss variants	Follows primary definition	PM4
PM5	Novel missense change at an amino acid residue where a different missense change determined to be pathogenic has been seen before	Refers to "ClinGen's RASopathy Expert Panel consensus methods for variant interpretations"	PM5(S): ⩾2 different pathogenic missense changes<br>PM5: 1 different pathogenic missense change
PP1	Co-segregation with disease in multiple affected family members in a gene definitively known to cause the disease	Refers to "ClinGen's RASopathy Expert Panel consensus methods for variant interpretations"	PP1(S): ⩾7 meioses<br>PP1(M): 5~6 meioses<br>PP1: 3~4 meioses
PP2	Missense variant in a gene that has a low rate of benign missense variation and in which missense variants are a common mechanism of disease	Follows primary definition and applicable for *DNMT1* (Z >3.09)	PP2
PP3	Multiple lines of computational evidence support a deleterious effect on the gene or gene product (conservation, evolutionary, splicing impact, etc.)	Follows primary definition	PP3
PP4	Patient's phenotype or family history is highly specific for a disease with a single genetic etiology	Clinical phenotypes linked to *WNK1/HSN2* , *NTRK1* , *SCN9A* , *DNMT1*, *RFC1*, *and NOTCH2NLC* are considered as highly specific	PP4
PP5	Reputable source recently reports variant as pathogenic but the evidence is not available to the laboratory to perform an independent evaluation	Currently, there are no resources that are acceptable for this criterion	Not applicable

Supplementary Table 3 Long-range flanking PCR and repeat-primed PCR protocols for detection of *RFC1* repeat expansions

Protocol	Primer (5'-3')	Reaction	Cycling
Long-range flanking PCR	Forward: TCAAGTGATACTCCAGCTACACCGTTGC<br>Reverse: CAGCATTGTGGGAGACAGGCCAATCACT	MiliQ 4.5 µl<br>2×Buffer 12.5 µl<br>dNTP 5 µl<br>Primer F (10pmol) 0.75 µl<br>Primer R (10pmol) 0.75 µl<br>KOD FX 0.5 µl<br>DNA 1.0 µl<br>Total 25 µl	95℃ 5min<br>95℃ 30s<br>65℃ 30s (30cycles: 95℃ 30s, 65℃ 30s, 72℃ 30s)<br>72℃ 30s<br>72℃ 10min<br>4℃ ∞
Repeat-primed PCR	**AAGGG**<br>Forward: [6FAM]TCAAGTGATACTCCAGCTACACCGT<br>Anchor: CAGGAAACAGCTATGACC<br>Reverse: CAGGAAACAGCTATGACCGGGAAGGGAAGGGAAGGGAA<br>**ACAGG**<br>Forward: [6FAM]TCAAGTGATACTCCAGCTACACCGT<br>Anchor: CAGGAAACAGCTATGACC<br>Reverse: CAGGAAACAGCTATGACCACAGGACAGGACAGGACAGG	MiliQ 1.76 µl<br>2×Buffer 7.5 µl<br>dNTP 3 µl<br>Primer F (10µM) 0.45 µl<br>Anchor(10µM) 0.45 µl<br>Primer R (5µM) 0.09 µl<br>DMSO 0.45 µl<br>KOD FX Neo 0.3 µl<br>DNA 1.0 µl<br>Total 15 µl	98℃ 3min<br>98℃ 10s<br>70℃ 15s (35cycles: 98℃ 10s, 70℃ 15s, 68℃ 4min)<br>68℃ 4min<br>68℃ 5min<br>4℃ ∞
	**AAAAG**<br>Forward: [6FAM]TCAAGTGATACTCCAGCTACACCGT<br>Anchor: CAGGAAACAGCTATGACC<br>Reverse1:<br>CAGGAAACAGCTATGACCAACAGAGCAAGACTCTGTTTCAAAAAAG<br>AAAAGAAAAGAAAAGAAAA<br>Reverse2:<br>CAGGAAACAGCTATGACCAACAGAGCAAGACTCTGTTTCAAAAAGA<br>AAAGAAAAGAAAAGAAAA<br>Reverse3:<br>CAGGAAACAGCTATGACCAACAGAGCAAGACTCTGTTTCAAAAGAA<br>AAGAAAAGAAAAGAAAA	MiliQ 1.58 µl<br>2×Buffer 7.5 µl<br>dNTP 3 µl<br>Primer F (10µM) 0.45 µl<br>Anchor(10µM) 0.45 µl<br>Primer R1(5µM) 0.09 µl<br>Primer R2 (5µM) 0.09 µl<br>Primer R3 (5µM) 0.09 µl<br>DMSO 0.45 µl<br>KOD FX Neo 0.3 µl<br>DNA 1.0 µl<br>Total 15 µl	98℃ 3min<br>98℃ 10s<br>70℃ 15s (35cycles: 98℃ 10s, 70℃ 15s, 68℃ 4min)<br>68℃ 4min<br>68℃ 5min<br>4℃ ∞

	**AAAGG**<br>Forward: [6FAM]TCAAGTGATACTCCAGCTACACCGT<br>Anchor: CAGGAAACAGCTATGACC<br>Reverse: CAGGAAACAGCTATGACCGGAAAGGAAAGGAAAGGAAA	MiliQ 1.76 µl<br>2×Buffer 7.5 µl<br>dNTP 3 µl<br>Primer F (10µM) 0.45 µl<br>Anchor (10µM) 0.45 µl<br>Primer R (5µM) 0.09 µl<br>DMSO 0.45 µl<br>KOD FX Neo 0.3 µl<br>DNA 1.0 µl<br>Total 15 µl	95℃ 5min<br>95℃ 45s<br>70℃ 30s -1℃/cycles<br>72℃ 50s<br>95℃ 45s<br>70℃ 30s -1℃/cycles<br>72℃ 50s<br>95℃ 45s<br>69℃ 30s -1℃/cycles<br>72℃ 50s<br>(above 9 steps: 13 cycles)<br>95℃ 45s<br>58℃ 30s<br>72℃ 50s<br>(above 3 steps: 35 cycles)<br>72℃ 10min<br>4℃ ∞

Supplementary Table 4 Interpretation of pathogenic/likely pathogenic variants using ACMG/AMP guidelines

Case NO.	Gene	Zygosity	Nucleotide	Protein	Pathogenicity	ACMG criteria	gnomAD	jMorp	PolyPhen2	SIFT	PROVEAN	FATHMM	Condel
P16,P19,P34,P35	*WNK1/HSN2*	Homozygous	c.3237dup	p.Asp1080*	P	PVS+PS4+PM2+PP4	0	0	/	/	/	/	/
P18	*WNK1/HSN2*	Compound heterozygous	c.3237dup	p.Asp1080*	P	PVS+PS4+PM2+PP4	0	0	/	/	/	/	/
			c.2615C>G	p.S872*	P	PVS+PS4(P)+PM2+PP4	0	0	/	/	/	/	/
P73	*WNK1/HSN2*	Compound heterozygous	c.3237dup	p.Asp1080*	P	PVS+PS4+PM2+PP4	0	0	/	/	/	/	/
			c.2971C>T	p.Arg991*	P	PVS+PS4(P)+PM2+PP1+PP4	0	0	/	/	/	/	/
P46	*NTRK1*	Compound heterozygous	c.1642del	p.R548Gfs	P	PVS+PS4+PM2+PM3+PP4	0	0	/	/	/	/	/
			c.2002G>T	p.D668Y	P	PS4+PM2+PM3+PP3+PP4	0	0	D	D	D	D	D
P47	*NTRK1*	Compound heterozygous	c.1642del	p.R548Gfs	P	PVS+PS4+PM2+PP4	0	0	/	/	/	/	/
			c.1786C>T	p.R596*	P	PVS+PS4(M)+PM2+PP4	0	0	/	/	/	/	/
P68	*NTRK1*	Compound heterozygous	c.1642del	p.R548Gfs	P	PVS+PS4+PM2+PP4	0	0	/	/	/	/	/
			c.2285C>T	p.P762L	LP	PS4(M)+PM2+PP3+PP4	0	0	D	D	D	D	D
P1, P2	*SCN9A*	Homozygous	c.3993delinsTT	p.Leu1331Phefs*	P	PVS+PS4(P)+PM2+PP1(S)+PP4	0	0	/	/	/	/	/
P41	*SCN9A*	Heterozygous	c.4895C>A	p.Ala1632Glu	P	PS3+PS4+PM2+PM5(S)+PM6+PP3+PP4	0	0	D	D	D	D	D
P5	*DNMT1*	Heterozygous	c.1706A>G	p.H569R	LP	PS4(P)+PM2+PP2+PP3+PP4	0	0	D	D	D	B	D
P24	*DNMT1*	Heterozygous	c.1619A>G	p.Y540C	P	PS4(P)+PM2+PM5(S)+PM6+PP2+PP3+PP4	0	0	D	D	D	B	D
P36	*NOTCH2NLC*	Heterozygous	(GGC)exp		P	PS3+PS4+PM2+PP4	/	/	/	/	/	/	/
P3,P4,P9,P21,P31,P42,P53,P67	*RFC1*	Homozygous	[(AAGGG)exp/(AAGGG)exp]		P	PS3+PS4+PP4	/	/	/	/	/	/	/
P10,P14,P15,P20,P22,P23,P48,P78	*RFC1*	Homozygous	[(ACAGG)exp/(ACAGG)exp]		LP	PS4+PM2+PP4	/	/	/	/	/	/	/
P32,P50,P60,P79	*RFC1*	Compound heterozygous	(AAGGG)exp		P	PS3+PS4+PP4	/	/	/	/	/	/	/
			(ACAGG)exp		LP	PS4+PM2+PP4	/	/	/	/	/	/	/

Supplementary Table 5 Clinical features of 20 cases with disease-associated *RFC1* repeat expansions

Case NO.	P3	P4	P9	P21	P31	P42	P53	P67	P10	P14	P15	P20	P22	P23	P48	P78	P32	P50	P60	P79
**Genotype**	[(AAGGG)exp/(AAGGG)exp]	[(AAGGG)exp/(AAGGG)exp]	[(AAGGG)exp/(AAGGG)exp]	[(AAGGG)exp/(AAGGG)exp]	[(AAGGG)exp/(AAGGG)exp]	[(AAGGG)exp/(AAGGG)exp]	[(AAGGG)exp/(AAGGG)exp]	[(AAGGG)exp/(AAGGG)exp]	[(ACAGG)exp/(ACAGG)exp]	[(ACAGG)exp/(ACAGG)exp]	[(ACAGG)exp/(ACAGG)exp]	[(ACAGG)exp/(ACAGG)exp]	[(ACAGG)exp/(ACAGG)exp]	[(ACAGG)exp/(ACAGG)exp]	[(ACAGG)exp/(ACAGG)exp]	[(ACAGG)exp/(ACAGG)exp]	[(AAGGG)exp/(ACAGG)exp]	[(AAGGG)exp/(ACAGG)exp]	[(AAGGG)exp/(ACAGG)exp]	[(AAGGG)exp/(ACAGG)exp]
**WES**	Y	Y	Y	Y	Y	Y	/	/	Y	Y	Y	/	/	Y	/	/	Y	/	/	/
**Family history**	S	AR	S	S	S	S	Con	S	Con	Con	Con	/	AR	Con	S	S	S	/	S	S
**Gender**	F	M	F	M	M	M	M	M	M	M	M	F	M	M	F	M	F	F	M	F
**Age**	Early 70s	Early 70s	Early 60s	Early 60s	Late 70s	Early 40s	Late 60s	Early 80s	Early 70s	Early 60s	Late 60s	Late 70s	Late 70s	Late 50s	Early 70s	Late 60s	Early 70s	Late 60s	Late 70s	Early 70s
**Onset**	Early 60s	Late 60s	Early 60s	Late 40s	Late 70s	Early 40s	Late 50s	Early 70s	Early 70s	Early 50s	Early 60s	Late 30s	Late 60s	Late 40s	Early 40s	Early 60s	Late 50s	Late 40s	Early 70s	Early 60s
**Original**	Standing/walking unsteadiness	Paresthesia	Walking unsteadiness	Dysuria	Walking unsteadiness	Paresthesia	Walking unsteadiness	Paresthesia	Gait disturbance	Numbness	Walking unsteadiness	Muscle cramp	Dysarthria	Numbness	Paresthesia	Numbness	Paresthesia	Numbness	Walking unsteadiness	Paresthesia
**Cough**	/	-	-	/	+	-	+	-	-	/	+	-	-	/	-	-	-	/	-	-
**Hearing loss**	/	Right	-	-	-	-	Mild	-	-	-	-	Mild	-	/	+	-	-	/	-	-
**Eye movement disorder**	-	Saccadic eye movement	-	Diplopia	Nystagmus	-	-	-	Gaze disturbance	-	Nystagmus; saccadic eye movement	-	Nystagmus; diplopia	/	Nystagmus; diplopia;strabismus	-	-	/	-	-
**Dysarthria /dysphagia**	Dysarthria	Mild dysphagia	-	Dysarthria	Dysarthria	-	Dysphagia	-	-	-	-	-	Dysarthria	/	Dysphagia; dysarthria	-	-	/	-	-
**Motor**	-	Interosseus muscle atrophy	-	-	-	-	-	Left hamstrings muscle weakness	Gastrocnemius muscle atrophy	-	-	Interosseus muscle weakness/atrophy	-	/	Interosseus muscle weakness	-	Flexion/extension weakness of shoulder and hip joint	-	Interosseus muscle weakness	-
**Pain sensation**	↓	↓	↓	↓	-	↓	-	↓	↓	↓	↓	↓	↓	↓	↓	-	↓	↓	↓	↓
**Vibration sensation**	↓	↓	↓	↓	↓	-	↓	↓	↓	↓	↓	↓	↓	-	↓	↓	↓	-	↓	↓
**Position sensation**	-	-	-	↓	-	-	-	↓	-	-	-	↓	↓	-	-	↓	↓	↓	-	-
**Dysautonomia**	-	Neurogenic bladder	/	Dysuria;constipation	Nycturia; dyshidrosis; orthostatic hypotension	-	/	Micturition; constipation	-	Constipation	-	Hypotonic bladder	-	/	Constipation;nycturia; orthostatic hypotention	Constipation	Orthostatic hypotension;nycturia;constipation; defecation syncope	-	Orthostatic hypotension	-
**Tendon reflex**	↑	↓	↓	↑	↓	-	/	↓	-	-	↓	↓	↓	/	↓	↑	↑	↓	↓	↓
**Ataxia**	+	-	-	+	+	-	+	-	-	+	+	+	+	-	+	-	+	-	+	-
**Romberg sign**	+	-	+	+	+	+	/	+	+	+	+	+	+	/	/	+	-	+	+	-
**Tremor**	/	-	-	-	+	/	/	-	+	-	-	+	-	/	-	-	+	/	+	-
**Other**	Left Chaddock (+)			Hyposmia;dysgeusia	Pakinsonism		Vestibular hypofunction	Pakinsonism								Dysgeusia				Dysgeusia
**Median SNCS**	NE	NE	SNAP ↓	SNAP ↓	NE	SNAP↓	NE	NE	NE	NE	NE	NE	NE	/	NE	NE	SNAP ↓	SNAP ↓	SNAP ↓	SNAP ↓
**Sural SNCS**	NE	NE	SNAP ↓	NE	NE	NE	NE	NE	NE	NE	NE	NE	NE	SNAP ↓	NE	NE	SNAP ↓	NE	NE	NE

**Motor nerves**	-	-	-	-	-	-	-	-	-	-	-	/	/	-	-	-	-	-	Tibial nerve	-
**F-Latency**	/	-	/	-	-	-	/	-	/	-	-	/	/	/	-	-	-	-	-	-
**F-Occurrence**	/	-	/	-	-	-	/	-	/	-	-	/	/	/	Tibial 70%	-	-	-	Median 31%; Ulnar 56%	-
**SEP**	NE	/	/	/	/	Upper and lower: prolonged latency	+	Lower: NE; Upper: prolonged latency	/	/	Lower: NE; Upper: prolonged latency	/	/	/	/	/	-	/	/	/
**MRI_atrophy**	Cerebellum; spinal cord	-	/	Cerebellum; spinal cord (mild)	Cerebellum	/	-	-	Cerebellum	-	Cerebellum	Cerebellum; cerebrum (Age-Related)	Cerebellum	/	-	Cerebellar	/	/	Frontal lobe	/
**SPECT**	/	/	/	Cerebellum ↓	/	/	/	/	/	Cortex multifocal ↓	Cerebellum ↓	-	/	/	Cerebellum ↓	/	/	/	/	/

Y: yes; S: sporadic; AR: autosomal recessive; Con: consanguineous parents; F: female; M: male; ↓ : decreased; ↑ : increased; +: positive; -: negative; /: no data; NE: not evoked;
SNCS: senosry nerve conduction study; SNAP: sensory nerve action potentials; MCV: motor nerve conduction velocity; CMAP: compound muscle action potential; SEP: somatosensory evoked potential
